# A comparison of the renal function biomarkers serum creatinine, pro-enkephalin and cystatin C to predict clearance of pemetrexed

**DOI:** 10.1007/s00280-024-04717-w

**Published:** 2024-10-04

**Authors:** N. de Rouw, R. Beunders, O. Hartmann, J. Schulte, R. J. Boosman, H. J. Derijks, D. M. Burger, M. M. van den Heuvel, L. B. Hilbrands, P. Pickkers, R. ter Heine

**Affiliations:** 1 Department of Pharmacy, Radboudumc, Nijmegen, The Netherlands; 2 Department of Pharmacy, Amphia Hospital, Breda, The Netherlands; 3 Department of Intensive Care Medicine, Radboudumc, Nijmegen, The Netherlands; 4 Radboud Center for Infectious Diseases, Radboudumc, Nijmegen, The Netherlands; 5 Radboud Institute Molecular Life Sciences, Radboudumc, Nijmegen, The Netherlands; 6 SphingoTec GmbH, Hennigsdorf, Germany; 7 Department of Pharmacy and Pharmacology, Antoni Van Leeuwenhoek-The Netherlands Cancer Institute, Amsterdam, The Netherlands; 8 Department of Pharmacy, Jeroen Bosch Ziekenhuis, Den Bosch, The Netherlands; 9 Department of Pulmonology, Radboud University Medical Center, Nijmegen, The Netherlands; 10 Department of Nephrology, Radboud University Medical Center, Nijmegen, The Netherlands

**Keywords:** Pemetrexed, GFR, Renal function, Biomarker, Chemotherapy, Pharmacokinetics, Lung cancer

## Abstract

**Introduction:**

For drugs with a narrow therapeutic window, there is a delicate balance between efficacy and toxicity, thus it is pivotal to administer the right dose from the first administration onwards. Exposure of pemetrexed, a cytotoxic drug used in lung cancer treatment, is dictated by kidney function. To facilitate optimized dosing of pemetrexed, accurate prediction of drug clearance is pivotal. Therefore, the aim of this study was to investigate the performance of the kidney function biomarkers serum creatinine, cystatin C and pro-enkephalin in terms of predicting the elimination of pemetrexed.

**Methods:**

We performed a population pharmacokinetic analysis using a dataset from two clinical trials containing pharmacokinetic data of pemetrexed and measurements of all three biomarkers. A three-compartment model without covariates was fitted to the data and the obtained individual empirical Bayes estimates for pemetrexed clearance were considered the “true” values (Cl_true_). Subsequently, the following algorithms were tested as covariates for pemetrexed clearance: the Chronic Kidney Disease Epidemiology Collaboration equation using creatinine (CKD-EPI_CR_), cystatin C (CKD-EPI_CYS_), a combination of both (CKD-EPI_CR-CYS_), pro-enkephalin as an absolute value or in a combined algorithm with age and serum creatinine, and lastly, a combination of pro-enkephalin with cystatin C.

**Results:**

The dataset consisted of 66 subjects with paired observations for all three kidney function biomarkers. Inclusion of CKD-EPI_CR-CYS_ as a covariate on pemetrexed clearance resulted in the best model fit, with the largest decrease in objective function (p < 0.00001) and explaining 35% of the total inter-individual variability in clearance. The predictive performance of the model to containing CKD-EPI_CR-CYS_ to predict pemetrexed clearance was good with a normalized root mean squared error and mean prediction error of 19.9% and 1.2%, respectively.

**Conclusions:**

In conclusion, this study showed that the combined CKD-EPI_CR-CYS_ performs best in terms predicting pharmacokinetics of pemetrexed. Despite the hypothesized disadvantages, creatinine remains to be a suitable and readily available marker to predict pemetrexed clearance in clinical practice.

## Introduction

For drugs with a narrow therapeutic window, there is a delicate balance between efficacy and toxicity. Therefore, it is pivotal to administer the right dose from the first administration onwards to ensure both safe and effective exposure. For renally cleared drugs, the systemic exposure directly relates to renal function [1]. Pemetrexed is a cytotoxic drug used for the treatment of lung cancer [2]. Similar to the cytotoxic drug carboplatin [3], it is known that renal function, besides dose, is the sole determinant for pemetrexed exposure and, thus, efficacy and toxicity [4–6].

Several biomarkers are available to predict the clearance of renally-excreted drugs. The gold standard would be the measurement of true glomerular filtration rate (GFR), however, this is an invasive procedure not routinely applied in clinical practice. Serum creatinine-based estimations, such as the Modification of Diet in Renal Disease (MDRD) [7] and Chronic Kidney Disease Epidemiology Collaboration (CKD-EPI) [8] equations, are the most commonly used in clinical practice and outperform the serum creatinine-based Cockgroft-Gault equation, which was developed for estimation of creatinine clearance [9]. Recognized disadvantages of creatinine are muscle mass-dependency, influences of diet, muscle activity and atrophy and tubular excretion and absorption. This may result in unreliable estimation of kidney function, especially in cancer patients, which are often underweight and sarcopenic [10]. Cystatin C is proven to be a suitable biomarker to estimate glomerular filtration rate, yet there are some conflicting studies on the reliability of serum cystatin C in cancer patients [11–14]. In some studies upregulation of cystatin C was reported [11, 13], with a risk for underestimating kidney function. Nonetheless, cystatin C was shown to be able to predict pharmacokinetics of the cytotoxic drugs topotecan and carboplatin [15, 16]. Proenkephalin (PENK) is a novel muscle-independent biomarker for kidney function in critically ill patients [17]. To date, PENK has not yet been evaluated to facilitate prediction of drug clearance, nor as a biomarker for kidney function in cancer patients.

The notion that dose individualization of anticancer drugs can improve treatment is gaining momentum, not only in the medical community, but also in cancer patients. A recent study showed that most metastatic breast cancer patients are willing to have drug dose adaption based on their individual characteristics, rather than receiving the standard dose [18]. To facilitate dose individualization of pemetrexed, accurate prediction of drug clearance is pivotal. Therefore, the aim of this study was to investigate the performance of various kidney function biomarkers and algorithms in terms of predicting the elimination of the renally cleared anti-cancer drug pemetrexed in lung cancer patients.

## Methods

### Dataset

A rich pharmacokinetic dataset, with at least 4 samples per patient and sampling based on a validated limited-sampling strategy [19] and all three kidney function biomarkers from two multicentre clinical trials were available (clinicaltrials.gov identifiers NCT03655821 and NCT03656549 [20, 21]). For each patient, the following information was incorporated in the dataset: pemetrexed dose, infusion duration, sampling times and plasma concentrations of pemetrexed, sex, age, weight, height, baseline serum creatinine, baseline serum cystatin C and baseline plasma PENK.

Weight and height were used to calculate body surface area (BSA, m^2^) and body mass index (BMI kg/m^2^). Using BMI, age and sex, the body fat percentage was calculated to subsequently assess fat-free mass index (FFMI kg/m^2^) [22]. The FFMI can be used as a measure for sarcopenia. Used cut-off values for sarcopenia for males and females were FFMI < 17 and < 15 kg/m^2^, respectively.

### Kidney function biomarkers

Serum creatinine was analyzed using validated assays implemented in routine care. Plasma samples to determine cystatin C and PENK were centrifugated and stored at − 40 °C within 1 h after collection at baseline. Cystatin C quantification was performed at three sites with different assays, all based on immunochemistric priniciple: nephelometric on the Attellica Neph 630 (Siemens Healthineers, Germany) or turbidimetric on the COBAS6000/8000 (Roche Diagnostics, Germany). PENK bioanalysis was performed using an immunoassay as previously described (SphingoTec GmbH, Hennigsdorf, Germany) [23].

### Statistical analysis

Non-linear mixed effects modelling was performed using the software package NONMEM v7.4.1 (Icon, Ireland). A previously developed three-compartment pharmacokinetic model without covariates was fitted to the data [5]. The obtained individual empirical Bayes estimates for pemetrexed clearance were considered the “true” values (Cl_true_).

Thereafter, upon visual inspection of the relationship between Cl_true_ and the kidney function biomarkers, they were investigated as covariate for clearance in the pharmacokinetic model. The following covariates were investigated: the Chronic Kidney Disease Epidemiology Collaboration (CKD-EPI) equation using creatinine (CKD-EPI_CR_ in mL/min), cystatin C (CKD-EPI_CYS_ in mL/min), and a combination of both (CKD-EPI_CR-CYS_ in mL/min) [9]_._ PENK was assessed as an absolute concentration (PENK_ABS_ in pmol/L) or in a combined algorithm with age and serum creatinine to estimate glomerular filtration rate (as proposed by Beunders et al. (eGFR_PENK-SCR_ in mL/min). The equation is as follows:

$${\text{eGFR}}\, = \,72.5*tanh(\left( {5.8{-}0.6*log10\left( {age} \right){-}1.3*log10\left( {creatinine} \right){-}1.1*log10\left( {PENK} \right){-}0.3} \right)\, + \,84.2)$$

This equation was developed on a rich dataset of 1354 patients with different etiology and both steady state and non-steady state kidney function [24]. Additionally, a model with a combination of both CKD-EPI_CYS_ and PENK_ABS_ as muscle mass-independent biomarkers as covariates for clearance was evaluated. All outcomes of eGFR were expressed in ml/min and, thus, adjusted for individual body surface area (BSA). The serum creatinine based Cockgroft-Gault equation for estimation of creatinine clearance was not considered for investigation, as CKD-EPI-equations for estimation of glomerular filtration are currently the gold standard in the clinic.

To assess if incorporation of any of the kidney function covariates resulted in a significant improvement in model fit compared to the base model without covariates, the decrease in objective function value (ΔOFV) was used to calculate the corresponding p-value. The ΔOFV arises from the sum of squared differences of the observations from the model prediction and follows a Chi-square distribution (thus a ΔOFV -3.84 corresponds with a p-value of 0.05 at 1 degree of freedom). Moreover, as the hypothesis is that covariates explain interindividual variability, the decrease in unexplained interindividual variability (IIV) in clearance compared to the base model was evaluated. Individual objective function values were assessed in the sarcopenic and non-sarcopenic subgroups to investigate if the muscle mass-independent biomarkers PENK_ABS_ and cystatin C were the drivers for an improved model fit, endorsing the previous mentioned hypothesis that muscle mass-indepent biomarkers could be better predictors in cancer patients. From the final covariate models, the obtained population estimates for the clearance parameters and covariate effects resulted in model-derived equations to predict pemetrexed clearance (defined as Cl_pred_). To assess performance of the covariate models in terms of predicting systemic pemetrexed clearance (Cl_pred_ versus Cl_true_), accuracy and precision were determined as mean percentage error (MPE %) and normalized root mean squared error (NRMSE %), respectively. Confidence intervals for MPE were calculated, as described by Sheiner et al. [25]. To calculate confidence intervals of the NRMSE, uncertainty was calculated according to the distribution-free approach of Faber [26].

## Results

### Dataset

The dataset consisted of 66 subjects with paired observations for all three kidney function biomarkers and 378 paired observations of time and pemetrexed plasma concentrations. Half of the population was male and the median [IQR] age was 65 [59–71] years. Baseline median [IQR] kidney function (CKD-EPI_CR_) was 97.0 [85.4–104.1] mL/min, with a minority of patients with an impaired kidney function (five patients with CKD-EPI_CR_ < 60 mL/min and four patients with PENK > 80 pmol/L [27], respectively). Based on the predefined criteria, 19 patients (28.4%) of the subset were considered sarcopenic. For all baseline characteristics see Table 1.

**Table 1:** Baseline characteristics of the subpopulation for all three kidney function biomarkers (n = 66)

Population	N = 66
Sex *n* [%] male	33 [50.0]
Age [years]	65 [59–71]
Weight [kg]	77.1 [63.7–84.8]
BSA [m^2^]	1.9 [1.7–2.0]
FFMI [kg/m^2^] Male Female	18.4 [18.0–19.5] 15.6 [14.7–16.2]
Sarcopenia n [%]	19 [28.4%]
CKD-EPI_CR_ (mL/min)	97.0 [85.4–104.1]
CKD-EPI_CYS_ (mL/min)	75.1 [59.8 –92.2]
CKD-EPI_CR-CYS_ (mL/min)	89.0 [73.3–99.5]
PENK_ABS_ (pmol/L)	37.3 [30.8–46.1]
eGFR_PENK-CR_ (mL/min)	112.5 [98.9–122.6]

Data are expressed as median [interquartile range, IQR] unless otherwise specified

*BSA* body surface area, *CKD-EPI*  chronic kidney disease–epidemiology collaboration equation, *CR*  creatinine, *CYS*  cystatin C, *PENK*  proenkephalin

### Analysis

Upon visual inspection of the relationship between the different covariates and Cl_true_ for all three CKD-EPI equations and the eGFR_PENK-CR_, an apparent linear covariate relationship with clearance was observed and subsequently tested as covariate in the base model. A non-linear relationship was visually observed between PENK_ABS_ and CL_true_ and, therefore, PENK_ABS_ was investigated as a covariate with a power and exponential function. The exponential function resulted in best improvement of model fit, as represented in the largest ΔOFV (data not shown). Hence, the exponential covariate function was used to describe the relationship between PENK and CL_true_. The equations with different covariates and corresponding estimates are presented in Table 2.

**Table 2:** Model-derived equations for the different covariate models

Covariate	Model-derived equation for Cl_pred_
CKD-EPI_CR_ (mL/min)	1.66 + (0.0358^.^ CKD-EPI_SCR_)
CKD-EPI_CYS_ (mL/min)	2.46 + (0.0344^.^ CKD-EPI_CYS_)
CKD-EPI_CR-CYS_ (mL/min)	1.89 + (0.038^.^ CKD-EPI_**CR-**CYS_)
PENK_ABS_ (pmol/L)	5.62^.^ e^(−0.0877*(PENK^_ABS_^/35))^
eGFR_PENK-CR_ (mL/min)	1.82 + (0.0295^.^ eGFR_PENK-SCR_)
CKD-EPI_CYS_ (mL/min) + PENK_ABS_ (pmol/L)	5.81^.^ e^(−0.0206*(PENK^_ABS_^/35)).^ (CKD-EPI_CYS_/75)^0.378^

The number 35 in the PENK_ABS_ equation reflects the median PENK concentration in the population. The number 75 reflects the median CKD-EPI_CYS_ in the population

*Cl*_*pred*_  model-predicted clearance, *CKD-EPI*  chronic kidney disease epidemiology collaboration, *CR*  creatinine, *CYS*  cystatin C, *PENK*  proenkephalin, *ABS*  absolute, *eGFR*  estimated glomerular filtration rate

**Fig. 1:**
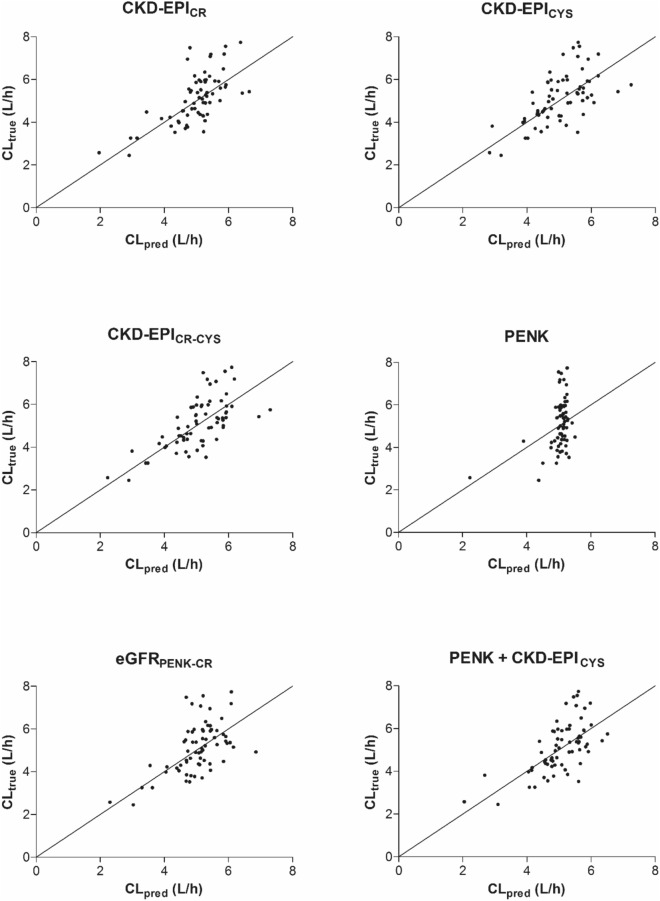
True clearance versus predicted clearance for all covariate models. Each dot represents an individual. The line represents the line of unity

In Fig. 1, the true clearance of each patient is plotted against the obtained value for Cl_pred_, together with the line of unity. A correlation between the studied biomarkers and pemetrexed clearance is observed for all covariate models. Visually, for PENK_ABS_, a higher proportional bias is observed around the line of unity compared to the other models.

The ΔOFV with corresponding p-values, unexplained interindividual variability as well as accuracy and precision (MPE% and NRMSE%) are presented in Table 3. Each covariate function resulted in a statistically significant improvement of the model and a decrease in IIV on clearance compared to the base model. The largest ΔOFV and decrease in IIV was observed for the model including CKD-EPI_CR-CYS_ as a covariate, indicating the best model fit (ΔOFV of − 49.5 and an IIV of 16.1%). Upon assessment of individual objective function values, there was no advantage of CKD-EPI_CYS_, PENK_ABS_ or of CKD-EPI_CYS_, + PENK_ABS_ in the subgroup of sarcopenic patients (data not shown). All models performed equally in terms of accuracy and precision. See Table 3 for all results on MPE% and NRMSE%.

**Table 3:** Predictive performance of covariate models

	IIV (%)	ΔOFV	*p*-value	MPE% [95% CI]	NRMSE % [95% CI]
*Base model*	24.8	Reference	Reference		
CKD-EPI_CR_ (mL/min)	16.2	− 38.1	< 0.00001	− 0.1 [− 4.0, 3.8]	17.0 [16.6, 17.3]
CKD-EPI_CYS_ (mL/min)	17.6	− 29.2	< 0.00001	0.81 [− 3.5, 5.1]	18.0 [17.7, 18.4]
CKD-EPI_CR-CYS_ (mL/min)	16.1	− 49.5	< 0.00001	1.2 [− 2.8, 5.1]	16.9 [16.6, 17.2]
PENK_ABS_ (pmol/L)	21.8	− 13.0	0.000311	2.8 [− 2.6, 8.2]	20.8 [20.6, 21.3]
eGFR_PENK-CR_ (mL/min)	18.1	− 32.8	< 0.00001	1.5 [− 2.8, 5.8]	18.6 [18.3, 19.0]
CKD-EPI_CYS_ (mL/min) + PENK_ABS_ (pmol/L)	17.3	− 41.0	< 0 .00001	1.7 [− 2.6, 5.9]	18.5 [18.2, 18.9]

Accuracy and precision are expressed as MPE and NRMSE, respectively. The presented p-value is the model improvement after incorporating the kidney function biomarkers as a covariate on pemetrexed clearance

*MPE*  mean prediction error, *NRMSE*  normalized root mean squared error, *CKD-EPI*  chronic kidney disease–epidemiology collaboration equation, *CR*  serum creatinine, *CYS*  cystatin C, *PENK*  proenkephalin, *ABS*  absolute, *eGFR*  estimated glomerular filtration rate, *CI* confidence interval

## Discussion

In this study we showed the potential of different kidney function biomarkers to predict renal clearance of drugs, specifically pemetrexed. The combined CKD-EPI_CR-CYS_ performed best in terms of model fit and explanation of variability. We hypothesized that in cancer patients that are often underweight and sarcopenic, a muscle mass-independent biomarker to predict clearance may be of added value to individualize dosing of renally excreted drugs. As creatinine is muscle-mass dependent, we estimated baseline sarcopenia in our population in terms of FFMI and found that approximately 28% had FFMI below the cut-off for sarcopenia [22]. Sarcopenia can result in overestimation of kidney function when a serum creatinine-based equation is used. In our population, median CKD-EPI_CR_ was higher than CKD-EPI_CYS_, possibly reflecting this effect. Although we found that adding a combination of PENK and cystatin C as covariates on clearance resulted in a good model fit, no specific advantage was attributable to the sarcopenic subgroup of the population.

Despite the disadvantages, generally creatinine performs well as a predictor of pemetrexed clearance. A hypothesis supporting the suitability of serum creatinine to predict pemetrexed pharmacokinetics is that both creatinine and pemetrexed are partly filtered and partly actively secreted by transporters in the kidney tubule [28–30]. Thus, both substances behave similarly in terms of elimination. Both, cystatin C and PENK, are freely filtered through the glomerulus [9, 17, 31], without further tubular handling, making these biomarkers reliable to accurately estimate glomerular filtration rate in general.

Although the evidence for cystatin C as a biomarker for kidney function in cancer patients is ambiguous, our study showed good predictive performance of the CKD-EPI_CR-CYS_ equation. Cystatin C was already shown to be superior to creatinine alone for the prediction of pharmacokinetics of the cytotoxic drug topotecan[15] in contrast to our findings for pemetrexed. Interestingly, renal elimination of topotecan is also hypothesized to be a combined process of filtration and active secretion [32, 33]. For the freely filtered carboplatin, two studies found the combined CKD-EPI_CR-CYS_ to be the best predictor for clearance [34, 35], in line with our findings for pemetrexed. Altogether, the role of the exact mechanisms of elimination of both the drugs and the biomarkers itself to predict clearance is ambiguous and there is no conclusive evidence on a clearly superior biomarker in terms of predicting drug clearance based on excretion mechanism.

One might argue that a limitation of this study is the limited number of patients with renal impairment and sarcopenia. To the best of our knowledge, this is the first study to evaluate PENK and drug clearance in cancer patients. The reported ‘normal’ range for PENK is up to 80 pmol/L (with a population median of 45 pmol/L in healthy subjects)) [27]. The median in our population was 37 pmol/L and, thus, comparable. PENK has especially shown potential in detecting acute changes in kidney function during acute kidney injury in critically ill patients [17, 27]. Our patient group only had few patients with moderate to severe renal impairment and thus a narrow interquartile range around the median. This reflects clinical practice, since pemetrexed is contraindicated for patients with a creatinine clearance < 45 ml/min. Further studies on PENK in cancer patients should include more patients with renal impairment. Moreover, it would be of interest to investigate a larger sarcopenic patient cohort.

Lastly, it should be noted that all tested algorithms were algorithms to estimate the glomerular filtration rate and that the true glomerular filtration rate in our population was unknown. We showed that irrespective of knowledge of the true glomerular filtration rate, the CKD-EPI_CR-CYS_ algorithm best predicted pemetrexed clearance. Prospective evaluation of using such an algorithm to dose to an established pharmacokinetic target, as proposed earlier [36], is warranted, investigating both pharmacokinetic and clinical endpoints.

In conclusion, this study showed that the combined CKD-EPI_CR-CYS_ performs best in terms predicting pharmacokinetics of pemetrexed. Moreover, despite the hypothesized disadvantages, creatinine remains to be a suitable marker, which can be easily applied as the assays are readily available in most clinics. Studies that include more patients with impaired kidney function and with drugs that are not actively secreted, but exclusively filtrated, like carboplatin, are needed to further investigate the use of PENK as a predictor for renal drug clearance in cancer patients and explore its use in dose optimization of cytotoxic drugs.

## Data Availability

All data are available upon reasonable request.
